# *Helicobacter pylori* Infection in Croatian Population: Knowledge, Attitudes and Factors Influencing Incidence and Recovery

**DOI:** 10.3390/healthcare10050833

**Published:** 2022-04-30

**Authors:** Pavle Vrebalov Cindro, Josipa Bukić, Dario Leskur, Doris Rušić, Ana Šešelja Perišin, Joško Božić, Jonatan Vuković, Darko Modun

**Affiliations:** 1 Department of Gastroenterology and Hepatology, University Hospital of Split, Spinčićeva 1, 21000 Split, Croatia; pcindro@mefst.hr (P.V.C.); jonatan.vukovic@mefst.hr (J.V.); 2 Department of Pharmacy, School of Medicine, University of Split, Šoltanska 2, 21000 Split, Croatia; jbukic@mefst.hr (J.B.); dleskur@mefst.hr (D.L.); drusic@mefst.hr (D.R.); dmodun@mefst.hr (D.M.); 3 Department of Pathophysiology, School of Medicine, University of Split, Šoltanska 2, 21000 Split, Croatia; jbozic@mefst.hr

**Keywords:** *Helicobacter pylori*, knowledge, attitudes, awareness, general population, Croatia

## Abstract

Basic and clinical knowledge about *Helicobacter pylori* infections has been improved in the past. However, the translation of this knowledge into public health intervention has remained poor. A survey based cross-sectional study was performed to assess the factors regarding the *H. pylori* infection in the general population. The survey was conducted using a previously developed questionnaire, adapted for the population in Croatia. Respondents (N = 1131) had a good knowledge score with a median of 4 out of 5 correct answers (interquartile range: 2–4). Senior participants had a lower frequency of high knowledge answers about *H. pylori* (43.1%) compared to younger (56.1%) and middle-aged participants (51.5%, *p* = 0.014). Rural participants had a higher frequency of low knowledge answers compared to urban and suburban ones (21.7% vs. 9.5% and 9.4%, *p* = 0.011). Only 315 participants (27.9%) were screened for the *H. pylori* infection, despite high support for the screening programs among the untested (74.7%) and tested (85.7%). Habits of smoking (*p* = 0.036) and coffee drinking (*p* = 0.008) were associated with more symptoms after eradication therapy. Further education is needed for the groups at risk for *H. pylori* infection, especially to raise the awareness of the importance of screening programs. More research is warranted to assess the effects of dietary changes on therapy outcomes.

## 1. Introduction

*Helicobacter pylori* (*H. pylori*) has been established as the most prevalent chronic infection globally, that, according to the previous studies, has affected more than a half of the world population [1]. Furthermore, this infection causes chronic gastritis in all of the infected patients, and could be a major cause of other diseases, such as a peptic ulcer disease, atrophic gastritis and gastric cancer. Moreover, there are data on the association between the *H. pylori* infection and irritable bowel syndrome [1,2,3,4,5]. It was also associated with certain non-gastrointestinal diseases, such as chronic kidney disease [6,7]. The high costs of this infection to health systems commands constant evaluation of *H. pylori* treatment and diagnosis [1,2,3]. Despite the fact that the prevalence of *H. pylori* has been decreasing in large countries such as the United States, Japan and Germany, the prevalence has remained high in the population which has a high incidence of gastric cancer. Moreover, *H. pylori* infection has been recognized as the most common infection-related cause of death by cancer. Basic and clinical knowledge on the matter of *H. pylori* has raised in the past, but the translation of this knowledge into public health intervention, such as population-wide screening and eradication, has remained poor [8,9].

Previous studies showed that it should be recommended that susceptibility tests of *H. pylori* are performed routinely in clinical practice [10,11,12]. Therefore, there is a need to improve *H. pylori* testing practice, which should be adapted to patients’ age, symptoms and drug utilization, provide adequate reliability and availability, and ensure low costs for health systems. Moreover, the majority of *H. pylori* therapies are utilized without an investigation of the prevalence of antibiotic resistance. One of the main reasons for this type of infection management is that susceptibility testing of *H. pylori* is still not commonly available [10,11,12]. Another problem of *H. pylori* management is a high rate of infection recurrence. Interestingly, a study by Zhao et al. [13] showed that European countries had the highest recurrence rate. Moreover, an increasing trend of recurrence in the past 10 years was observed, which made it a great public health problem worldwide [13].

Despite the numerous problems addressed to the *H. pylori* infection, public awareness on this matter remains low. The results of the study by Teng et al. revealed that awareness of *H. pylori* infection is lacking [14]. However, the results of the same study showed a high acceptance rate of the screening tests. Similar results of insufficient *H. pylori* awareness were found in the study conducted by Hafiz et al. [15]. Moreover, the authors concluded that educational programs are recommended for improving patients’ awareness and knowledge about *H. pylori* infection [14,15]. A study conducted on patients with the peptic ulcer disease showed that health education contributed to the improvement of patients’ self-care ability and resulted in the reduction of *H. pylori* infection rates [16]. Based on these positive results, it seems reasonable that numerous health education studies conducted on patients would be feasible in the future.

Early detection of *H. pylori* decreases the risk of disease complications in patients. Moreover, the appropriate pharmacotherapy intervention leads to the eradication of this infection. In order to detect and eradicate this infection, the general population should be knowledgeable about basic information on *H. pylori* infection. Therefore, the aim of this study was to assess the knowledge, attitudes, habits and other factors regarding the *H. pylori* infection in the general population in Croatia.

## 2. Materials and Methods

### 2.1. Study Design and Population

This study was designed as a survey based cross-sectional study and approved by the University of Split School of Medicine Ethics Committee. The study was carried out from 1 April to 31 May 2021 in Croatia. The eligible participants were all Croatian citizens over the age of 18 years, not working as health care professionals. The survey, available as a Google form document, was distributed through a network of family physicians in Croatia. Prior to accessing the survey questionnaire, participants had to give an informed consent.

### 2.2. Survey Questionnaire

The survey used in this study was developed by Wu et al. [17] and adapted to the general population in Croatia (Supplementary File S1). Firstly, the original survey was translated into the Croatian language and then translated back to the English language by a native English speaker. A working group of pharmacologists and gastroenterologists reviewed each original survey item and chose the appropriate items for this study. Prior to the survey distribution, a pilot study was conducted among 15 non-health professional employees of the University of Split School of Medicine, in order to ensure the clarity of the survey and its suitability for the general population. Furthermore, the pilot study enabled the evaluation of the time needed to complete the survey, which was approximately 15 min.

The survey used in this study consisted of four sections and 34 items in total. The first section, consisting of 7 items, gathered demographic information of the participants, such as age, gender, education, place of residence, profession and lifestyle habits. The second part of the survey included 5 items measuring general knowledge on *H. pylori*. The third section, comprised of 9 items, gathered attitudes about *H. pylori* and sources of information used on this matter. The last section included information about personal experience with *H. pylori* infection and consisted of 13 items. The full text of the survey is available on request from the corresponding author.

### 2.3. Statistical Analysis

The respondents were classified into three groups based on the number of correct answers—a method based on the study by Wu et al. [17]. The low knowledge group answered all the questions either incorrectly or with “don’t know”, the moderate knowledge group had one to three correct answers, whereas the high knowledge group participants had four or five correct answers.

Association of *H. pylori* infection with participants’ demographic data and habits was determined. Moreover, the association of estimated symptoms’ improvement post treatment with previously mentioned data, as well as information about adverse drug reactions and attendance of follow-up examination were investigated. Fisher’s exact test with Cramer’s V measurement were used as a measure of association. Association was considered weak for Cramer’s V values below 0.1, moderate for values between 0.11 and 0.31, and strong for values over 0.31.

Data were presented as overall number and proportion (%) or median and interquartile range (IQR), where applicable. Data were analyzed using a chi-square test. Results were considered statistically significant for *p* < 0.05. Statistical analysis was conducted using SPSS (version 16.0, IBM Corporation, Chicago, IL, USA).

## 3. Results

### 3.1. Demographic Data

A total of 1131 people participated in the survey. Most of the included respondents were young (19–34 years) (43.5%) or middle-aged (35–60 years) (46.9%) and living in urban areas (77.2%). A majority were highly educated, with bachelor’s degrees (14.0%) or master’s degrees and above (52.8%). Moreover, a large proportion had a secondary educational level (30.2%). According to their occupation, most participants were Science and engineering professionals (24.3%) or classified as Other (32.0%). The larger part (83.1%) of participants were familiar with *H. pylori* bacteria. A total of 315 participants (27.9%; 23.3–30.5% 95% confidence interval (CI)) were tested for *H. pylori* infection with 156 participants (13.8%; 11.8–15.8% 95% CI) confirming they were positive. Full demographic data are presented in Table 1.

In regard to personal habits correlated to *H. pylori* infection, 951 (84.1%) participants had at least one aforementioned habit, with long-term consumption of coffee (46.1%) and sweets (47.2%) being the most prevalent (Table 1). The median number of lifestyle habits was 1 (IQR: 1–2).

### 3.2. H. pylori Knowledge

Participants had a median of four correct answers (IQR: 2–4). All answers were correctly marked by 15.6% of participants, while 11.5% had no correct answers. Overall, respondents had a good knowledge, with just over a half (52.7%) answering four or five out of five questions correctly.

Variation in knowledge between different demographic groups was noticed (Table 2). Senior participants, over the age of 60, had lower frequency of high knowledge about *H. pylori* (43.1%) compared to younger and middle-aged participants (56.1% and 51.5%, respectively, *p* = 0.014). Further, men had a higher proportion of low knowledge respondents in comparison to women (22.6% vs. 7.9% women, *p* < 0.001). Rural participants had higher frequency of low knowledge answers compared to urban and suburban ones (21.7% vs. 9.5% urban and 9.4% suburban, *p* = 0.011). There was also a difference in regard to participants’ occupation, with Students and Science and engineering professionals having more of the high knowledge respondents and less of the low knowledge respondents compared to Clerical support workers, Service and sales workers, or Skilled agricultural, forestry and fishery workers (Table 2, *p* < 0.001). Group of participants previously screened for *H. pylori* infection had more respondents with high knowledge in comparison to the group of participants without infection testing (Table 2, *p* < 0.001).

### 3.3. Sources of Information on H. pylori

The most commonly used sources of information on *H. pylori* were the Internet and social media (31.1%), with friends and family being in second place with 28.8%. Books were used by 250 participants (22.1%), while unspecified other sources were used by 17.8% of respondents. Only 20.8% of participants answered that they obtained their information on *H. pylori* during their medical examinations. Interestingly, TV and radio were only used by 4.4%, while no participants used newspapers and magazines as a source of information, reflecting changes in modern media consumption.

### 3.4. Attitudes about H. pylori Testing and Screening Programs

Most of the participants (70.6%) were not screened for the *H. pylori* infection, with the most common reason for not participating in screening being the lack of any symptoms (47.2%) and the test not being included in regular health examination (22.0%). Nevertheless, despite the low number of participants tested, support for screening was high among the untested, with 597 (74.7%) in support, 11 (1.4%) in opposition and 187 (23.4%) with no opinion (Table 3).

The support for screening programs was also high among the tested participants, with 85.7% in favor. More than half of the tested participants (58.7%) would suggest testing for the other members of their household. The majority would enter the treatment to eradicate the bacteria (89.2%) and would suggest the same for their family members in cases where they had been tested positive (88.6%) (Table 3).

### 3.5. H. pylori Eradication Therapy

A total of 156 participants (13.8%) had confirmed *H. pylori* infection. Most received eradication therapy (95.5%), while the rest were either not treated or were unsure if they received treatment (Table 4). The stated reason for not receiving treatment by the first participant was a belief that *H. pylori* infection had no effect on health and quality of life. The second claimed he was worried about adverse drug reactions and also chose an unspecified other reason for avoiding therapy. The third participant did not state his reasons.

Most of the treated participants were given antibiotics (94.6%) in a standard combination triple or quadruple therapy (83.2%). An alarming proportion of patients (23.5%) did not go to a follow-up appointment after the treatment. Furthermore, 15.4% were reinfected after the treatment and 19.5% were unsure whether they were reinfected. Treatments’ adverse drug reactions were experienced by 41.6%, with abdominal pain and dry mouth being the most common. There was a large proportion of participants (74.5%) who estimated symptoms’ change after the therapy as an Improvement or Complete improvement. Only one participant (0.7%) claimed that treatment worsened his symptoms. Treatment caused worry all the time or most of the time in little less than 10% of participants whereas most claimed that treatment caused them to worry sometimes (32.2%) or never (34.9%). Other treatment options due to unsatisfactory results were considered sometimes to all the time by 41.6% of participants (Table 4).

Despite almost three quarters of participants reporting symptoms improvements after the treatment, many still experienced a variety of symptoms as well as other difficulties after treatment (Table 5). The difficulties often experienced by the participants were heartburn, belching or flatulence, and fullness of stomach. Participants often complained they had to change their eating habits due to illness, including giving up their favorite food.

### 3.6. Factors Influencing Incidence of H. pylori Infection and Estimated Symptoms’ Improvement after the Treatment

*H. pylori* infection was moderately associated with the age, sex and profession of the participants (Table 6). Older participants had a higher incidence of infections than middle-aged and younger participants (27.9% vs. 18.4% middle-aged and 6.7% younger, *p* < 0.001). There was a larger proportion of female participants without infection (74.1% vs. 62.4% male, *p* < 0.001), even though the proportions of those infected were comparable (14.0% vs 14.9% male). This difference was likely influenced by a larger proportion of men answering they were unsure if they had an infection. Chief executives, senior officials and legislators (20.4%), Clerical support workers, service and sales workers (24.4%), and Plant and machine operators, and assemblers (25.0%) had the highest incidence of *H. pylori* infection, while Skilled agricultural, forestry and fishery workers and Students had the highest proportions of non-infected (100.0% and 83.3% Students, *p* < 0.001). Among the habits associated with infection, only alcohol consumption had a weak association (*p* = 0.041).

Estimated symptoms improvement after the treatment was associated with sex and certain habits (Table 6). Women had lower frequency of no change in symptoms (5.4% vs. 19.4% male, *p* = 0.009) as well as higher frequency of the Improvement answer (53.6% vs. 25.0% male), even though men had a higher proportion of Complete improvement (38.9% vs. 24.1% female). The aforementioned association was defined as moderate. Another moderate association was observed between coffee consumption and symptoms improvement, as participants who did not regularly consume coffee had better symptoms improvement, with 81.5% of non-consumers having improved or completely improved symptoms in comparison to 65.5% of coffee consumers (*p* = 0.008). Non-smoking was also moderately associated with better symptoms improvement as 78.0% of non-smokers had improved or completely improved symptoms in comparison to 64.1% of smokers (*p* = 0.036). A strong association was found with tea consumption, but those results had no validity as the sample of tea drinkers treated for *H. pylori* infection was small (N = 6).

## 4. Discussion

Overall, the knowledge of *H. pylori* of the included participants could be classified as very good. However, scores were significantly lower among the older population, above the age of 60, and among participants from rural areas. Both of those factors were previously recognized as *H. pylori* infection risk factors [18,19]. The physical health of the rural elderly was previously found to be strongly affected by their education and living conditions [20]. Educational interventions to improve the knowledge on the matter, as well as to promote general health literacy, is therefore necessary [14,21,22]. Nonetheless, health education of the elderly as well as those living in the rural areas has proven challenging, mostly owing to the lack of educational opportunities, remoteness, unfamiliarity with modern technologies and age-related cognitive decline [23,24]. Another interesting finding was that a lower level of knowledge resulted in almost no screening amongst participants, while those screened had higher levels of knowledge. This indicates that knowledge might be directly linked to participants’ willingness to participate in screening which is another argument in favor of the necessity of educational programs aimed towards the general population.

Most of the participants included in the present study did not undergo *H. pylori* screening mainly due to the lack of symptoms, which is problematic as the infection is usually asymptomatic. Hence, despite the high knowledge score, a number of participants thought they did not need to be tested because they lacked any symptoms. On the plus side, large proportions of both untested and tested participants supported *H. pylori* screening. This discrepancy between the number screened and support for screening was apparent. Other studies also showed high support for screening regardless of the number of participants actually screened [14,17]. Another positive was high support for eradication therapy for both their family members and for themselves, in cases where they were infected. This is further supported by the fact that almost every participant that was positive for *H. pylori* infection received the treatment. Only 13.8% of participants claimed to be infected with *H. pylori*, which was significantly lower than the estimated prevalence of infection in Croatia which was 52.7% [3].

Almost a third of all treated participants claimed their therapy lasted for more than 14 days, which could mean their original therapy failed and they had to take a re-treatment. A large study on the European Registry on *H. pylori* management (Hp-EuReg), conducted until 2018, found that physicians in the southeastern region, which included Croatia, predominantly prescribed the seven-day treatment regimens [25]. However, the same study found that recently there was a shift towards a longer duration of the treatment, in concordance with the new guidelines, with the goal of improving eradication rates [25,26]. In our study, an equal proportion of participants received the 7–10 days’ and 11–14 days’ therapies. Our results could reflect those changes in treatment, as the number of participants receiving longer treatment increased during the three-year gap between the two studies [25,26]. About 15% of those treated claimed to have had a recurrence of infection. It was unclear if it had been caused by reinfection after a successful eradication or by treatment failure [27]. Furthermore, 15% of participants strongly considered the alternative treatment options due to the unsatisfactory results of the original therapy. These numbers, combined with the number of participants who had treatment longer than 14 days, could be indicative of unfavorable treatment outcomes for certain participants.

One of the reasons could be antimicrobial resistance, as Croatia had a high rate of resistance to the antibiotics used in *H. pylori* treatment, especially clarithromycin [26]. This could limit the effectiveness of the triple therapy regimens, which used to be the predominant regimens in this region of Europe, even though quadruple regimens became the treatment of choice lately as these regimens had more success in the event of clarithromycin resistance [25,26].

Another obstacle for the successful eradication of *H. pylori* infection in Croatia was poor knowledge and the implementation of the Maastricht V/Florence consensus report guidelines among Croatian family physicians and medical students [28]. Furthermore, there was a poor correlation of the drug packs available on the Croatian market with treatment guidelines for *H. pylori*, which would lead to more leftover antibiotics and influence patient adherence [29]. These factors could have a deleterious effect on the development of antimicrobial resistance and need to be addressed in the future.

Among the participants in this study, risk factors for development of *H. pylori* infection were age over sixty years, male sex, being employed in certain professions and alcohol consumption. As no *H. pylori* screening was conducted as a part of this study, given the usually asymptomatic nature of the infection and an average Croatian infection rate that is higher than the one in this study, it is possible that many more participants were infected. Moreover, it is likely that those who tested positive were ones that had symptoms and that was the reason they were screened in the first place, especially since the most of untested participants declined screening because they lacked any symptoms. Based on those assumptions, we could argue that our risk factors were not actually risks of the infection’s development but risks of worsening of the infection and symptoms’ development.

Socio-economic factors, occupational risk factors and lifestyle factors, such as diet and smoking, were previously associated with the incidence of *H. pylori* infection. The same factors also presented as risk factors for the development of gastritis, stomach cancer and other complications [30,31]. In this study, we tried to investigate whether some of those factors could influence the recovery after treatment, defined as an improvement of the symptoms. The most interesting, albeit unsurprising, finding was that smoking and coffee consumption had a detrimental effect on recovery after treatment. Less clear effects were seen with tea consumption and participants’ sex, as these results were either conflicting or based on an extremely small sample. A study by Kang et al. [32] also found that certain dietary habits were related to improvements after eradication therapy. Spicy and salty food were found to be related to improvements of gastric atrophy and metaplasia, even though the authors of the study were not sure if those results were a consequence of their reduced intake or the fact that eradication of bacteria reduced their harmful effects [32].

As *H. pylori* infection presents a significant threat to global health, it is imperative to mitigate its burden with prevention and early detection, for which a broader participation in screening programs is necessary. This study showed that people who knew more about the disease and its complications were more likely to participate. Moreover, results showed that most participants used the Internet and social media as sources of information. Therefore, a nationwide educational campaign, with more presence on those media platforms, might be beneficial. So far, no such campaign has been conducted in Croatia.

The present study has several limitations. Firstly, the survey was conducted online which could limit the representativity of the sample as only those with sufficient digital literacy and with Internet access could participate in the study. Secondly, it was a cross-sectional study which provided only an observation and limited determination of causality. Next, the study relied on participants to accurately recollect the information, thus increasing the chance of recall bias. Furthermore, there was a difference in male and female population and unequal sample sizes could influence the results of the statistical analysis and introduce bias. However, a significant effect on the results is not expected due to the statistical tests that were used, that took the sample size into account and the data were compared as frequencies instead of absolute numbers. Another possible sample bias was the discrepancy between the number of highly educated people between our sample and the general population in Croatia. As the sample contained more people with university degrees, the results might be biased towards the higher knowledge score and more favorable attitudes towards screening and other healthcare interventions, which limits the generalizability of the findings.

## 5. Conclusions

The Croatian population had a relatively good knowledge of *H. pylori*, with the exception of the elderly, over the age of 60, and those living in a rural setting. The number of participants screened for *H. pylori* was subpar despite high support for national screening programs. More education is needed for the at risk groups, such as rural residents and senior citizens, and for raising the awareness of the importance of screening programs. Further research is warranted to assess the effects of dietary changes on therapy outcomes.

## Figures and Tables

**Table 1:** Demographic characteristics of participants (N = 1131).

Characteristics	N	%
Age		
19–34 years	492	43.5
35–60 years	530	46.9
>60 years	109	9.6
Sex		
Female	860	76.0
Male	270	23.9
Did not answer	1	0.1
Education level		
Primary education (and below)	6	0.5
Secondary education	342	30.2
Specialty	27	2.4
Bachelor’s degree	158	14.0
Master’s degree (and above)	597	52.8
Did not answer	1	0.1
Residence		
Urban	873	77.2
Suburban	149	13.2
Rural	92	8.1
Did not answer	17	1.5
Occupation		
Chief executives, senior officials and legislators	54	4.8
Science and engineering professionals	275	24.3
Technicians and associate professionals	86	7.6
Clerical support workers, service and sales workers	141	12.5
Skilled agricultural, forestry and fishery workers	4	0.4
Plant and machine operators, and assemblers	6	0.5
Student	203	17.9
Other	362	32.0
Habits		
Long-term alcohol	90	8.0
Long-term smoking	266	23.5
Long-term high-fat diet	245	21.7
Long-term sweets	534	47.2
Long-term seafood	78	6.9
Long-term preserved food	37	3.3
Long-term coffee	521	46.1
Long-term tea	52	4.6
Participants heard of *H. pylori*	940	83.1
Participants screened for *H. pylori* infection	315	27.9
Participants with *H. pylori* infection		
Yes	156	13.8
No	784	69.3
Do not know	158	14.0

**Table 2:** Factors affecting knowledge of *H. pylori*.

	Low (%)	Moderate (%)	High (%)	*p* *
Overall	130 (11.5)	405 (35.8)	596 (52.7)	
Age				
19–34 years	64 (13.0)	152 (30.9)	276 (56.1)	0.014
35–60 years	55 (10.4)	202 (38.1)	273 (51.5)	
>60 years	11 (10.1)	51 (46.8)	47 (43.1)	
Sex				
Female	68 (7.9)	310 (36.0)	482 (56.0)	<0.001
Male	61 (22.6)	95 (35.2)	114 (42.2)	
Education level				
Primary education (and below)	1 (16.7)	2 (33.3)	3 (50.0)	0.238
Secondary education	46 (13.5)	129 (37.7)	167 (48.8)	
Speciality	5 (18.5)	12 (44.4)	10 (37.0)	
Bachelor’s degree	12 (7.6)	55 (34.8)	91 (57.6)	
Master’s degree (and above)	66 (11.1)	206 (34.5)	325 (54.4)	
Residence				
Urban	83 (9.5)	317 (36.3)	473 (54.2)	0.011
Suburban	14 (9.4)	52 (34.9)	83 (55.7)	
Rural	20 (21.7)	34 (37.0)	38 (41.3)	
Occupation				
Chief executives, senior officials and legislators	5 (9.3)	24 (44.4)	25 (46.3)	<0.001
Science and engineering professionals	29 (10.5)	81 (29.5)	165 (60.0)	
Technicians and associate professionals	7 (8.1)	34 (39.5)	45 (52.3)	
Clerical support workers, service and sales workers	19 (13.5)	59 (41.8)	63 (44.7)	
Skilled agricultural, forestry and fishery workers	2 (50.0)	2 (50.0)	0 (0.0)	
Plant and machine operators, and assemblers	2 (33.3)	2 (33.3)	2 (33.3)	
Student	19 (9.4)	51 (25.1)	133 (65.5)	
Other	47 (13.0)	152 (42.0)	163 (45.0)	
Participants screened for *H. pylori* infection				
Yes	1 (0.3)	103 (32.7)	211 (67.0)	<0.001
No	115 (14.4)	300 (37.5)	384 (48.1)	

* Fisher’s exact test. Knowledge levels stratified according to number of correct answers: low (all answers incorrect or did not know), moderate (1–3 correct answers) and high (4–5 correct answers).

**Table 3:** Attitudes towards *H. pylori* testing and therapy.

Participants Screened for *H. pylori* Infection	N	%
Yes	315	27.9
No	799	70.6
Did not answer	17	1.5
Questions for the patients who **were not** screened for *H. pylori*		
**Reason for not being screened**		
It is not included in hospital physical examination	176	22.0
There are no obvious symptoms and I don’t want to check them	377	47.2
I am young and not necessary to test it	114	14.3
I am old and worry about the risk of screening	6	0.8
Other	119	14.9
Did not answer	7	0.9
**Support for *H. pylori* screening**		
Yes	597	74.7
No	11	1.4
Neutrality	187	23.4
Did not answer	4	0.5
Questions for patients who **were** screened for *H. pylori*		
**Which of the following methods would you choose to screen for *Helicobacter pylori*?**		
Go and test *H. pylori* on my own	101	32.1
Routine medical examinations when available	181	57.5
Not clear	23	7.3
Did not answer	10	3.2
**If you are infected with *Helicobacter pylori*, would you be advised to screen your family for *Helicobacter pylori*?**		
Yes	185	58.7
No	52	16.5
Neutrality	72	22.9
Did not answer	6	1.9
**What is your attitude towards *Helicobacter pylori* screening?**		
No need to check	4	1.3
Neutrality	36	11.4
Supporting screening	270	85.7
Did not answer	5	1.6
**If you are infected with *Helicobacter pylori*, will you eradicate it?**		
Yes	281	89.2
No	5	1.6
Neutrality	22	7.0
Did not answer	7	2.2
**If you are negative for *Helicobacter pylori* but your family is positive for *Helicobacter pylori*, do you recommend your family to eradicate it?**		
Yes	279	88.6
No	4	1.3
No opinion	31	9.8
Did not answer	1	0.3

**Table 4:** *H. pylori* treatment.

Participants with *H. pylori* Infection	N	%
Yes	156	13.8
No	784	69.3
Not clear	158	14.0
Did not answer	33	2.9
**If you had an infection, did you receive a treatment?**		
Yes	149	95.5
No	3	1.9
Not clear	4	2.6
Did not answer	0	0.0
**Reasons for not receiving treatment**		
Carrying *H. pylori* has no impact on health and life	1	
Economic factors	0	
Worry about side effects of drug	1	
Fear of relapse after eradication	0	
Other	1	
Did not answer	1	
**Questions for patients who received treatment**		
**Treatment regimen included antibiotic**		
Yes	141	94.6
No	1	0.7
Not clear	7	4.7
Did not answer	0	0.0
**Received triple/quadruple eradication treatment**		
Yes	124	83.2
No	10	6.7
Not clear	15	10.1
Did not answer	0	0.0
**Duration of the treatment**		
<7 days	3	2.0
7–10 days	38	25.5
11–14 days	37	24.8
>14 days	45	30.2
Not sure	24	16.1
Did not answer	2	1.3
**Re-examination after eradication treatment**		
Yes	106	71.1
No	35	23.5
Not sure	8	5.4
Did not answer	0	0.0
**Reinfected after the treatment**		
Yes	23	15.4
No	96	64.4
Not sure	29	19.5
Did not answer	1	0.7
**Have you had any of the following adverse reactions during taking the medicine?**		
Abdominal pain	31	20.8
Diarrhea	14	9.4
Dry mouth	20	13.4
Constipation	10	6.7
Other	21	14.1
No adverse drug reactions	87	58.4
**Estimate symptoms’ improvement after the treatment**		
Deterioration	1	0.7
No change	13	8.7
Slight improvement	24	16.1
Improvement	70	47.0
Complete improvement	41	27.5
Did not answer	0	0.0
**Worry caused by treatment**		
All the time	5	3.4
Most of the time	9	6.0
Sometimes	48	32.2
Occasionally	35	23.5
Never	52	34.9
Did not answer	0	0.0
**I considered other treatment options due to unsatisfactory results of the original therapy**		
All the time	13	8.7
Most of the time	10	6.7
Sometimes	39	26.2
Occasionally	29	19.5
Never	57	38.3
Did not answer	1	0.7

**Table 5:** Symptoms and difficulties experienced post treatment.

Do You Still Have the Following Symptoms after Treatment?	Very Often (%)	Often (%)	Sometimes (%)	Almost Never (%)	Never (%)	Did Not Answer (%)
Abdominal pain regardless of intensity	9 (6.0)	8 (5.4)	46 (30.9)	17 (11.4)	17 (11.4)	52 (34.9)
Stomach (upper abdomen) fullness	14 (9.4)	22 (14.8)	42 (28.2)	9 (6.0)	15 (10.1)	47 (31.5)
Belching or flatulence	20 (13.4)	30 (20.1)	44 (29.5)	7 (4.7)	6 (4.0)	42 (28.2)
Vomiting	2 (1.3)	0 (0.0)	19 (12.8)	27 (18.1)	35 (23.5)	66 (44.3)
Nausea	7 (4.7)	13 (8.7)	44 (29.5)	18 (12.1)	19 (12.8)	48 (32.2)
Heartburn	15 (10.1)	23 (15.4)	44 (29.5)	18 (12.1)	10 (6.7)	39 (26.2)
Bitter taste	11 (7.4)	14 (9.4)	18 (12.1)	23 (15.4)	26 (17.4)	57 (38.3)
Lack of appetite	6 (4.0)	8 (5.4)	17 (11.4)	27 (18.1)	31 (20.8)	60 (40.3)
You must give up eating some favorite food due to illness	19 (12.8)	16 (10.7)	24 (16.1)	18 (12.1)	23 (15.4)	49 (32.9)
Be dissatisfied with your life	3 (2.0)	7 (4.7)	22 (14.8)	29 (19.5)	27 (18.1)	61 (40.9)
Situation affecting the continuation of daily amateur activities	8 (5.4)	4 (2.7)	19 (12.8)	24 (16.1)	33 (22.1)	61 (40.9)
The relationship with your relatives and friends is affected due to illness	3 (2.0)	4 (2.7)	10 (6.7)	31 (20.8)	40 (26.8)	61 (40.9)
Restrictions on sexual life	3 (2.0)	2 (1.3)	12 (8.1)	30 (20.1)	39 (26.2)	63 (42.3)
Insomnia	8 (5.4)	19 (12.8)	17 (11.4)	19 (12.8)	30 (20.1)	56 (37.6)
Illness has forced you to adopt a separate diet	27 (18.1)	17 (11.4)	25 (16.8)	19 (12.8)	16 (10.7)	45 (30.2)
Feel sad because of your illness	10 (6.7)	10 (6.7)	16 (10.7)	24 (16.1)	32 (21.5)	57 (38.3)
Frustrated by your illness	10 (6.7)	9 (6.0)	18 (12.1)	22 (14.8)	35 (23.5)	55 (36.9)
Feel nervous or afraid due to illness (such as fear of canceration, etc.)	8 (5.4)	12 (8.1)	16 (10.7)	25 (16.8)	32 (21.5)	56 (37.6)

**Table 6:** Factors influencing incidence of *H. pylori* infection.

	Factors Influencing Incidence of *H. pylori* Infection			Factors Influencing Estimated Symptoms’ Improvement Post Treatment		
	Fisher’s Exact Test	Cramer’s V	*p*	Fisher’s Exact Test	Cramer’s V	*p*
Age	47.730	0.146	**<0.001**	11.644	0.192	0.204
Sex	18.653	0.135	**<0.001**	13.187	0.301	**0.009**
Residence	2.243	0.033	0.676	3.635	0.088	0.971
Occupation	48.631	0.149	**<0.001**	34.070	0.24	0.083
Education level	5.838	0.046	0.791	14.340	0.175	0.327
Experienced treatment adverse drug reactions	n/a	n/a	n/a	3.459	0.154	0.48
Follow-up medical examination after the treatment	n/a	n/a	n/a	3.074	0.087	0.974
Habits						
Seafood	0.620	0.021	0.781	1.145	0.028	0.998
High-fat diet	5.271	0.07	0.069	1.927	0.111	0.77
Coffee	0.276	0.016	0.871	13.689	0.304	**0.008**
Preserved food	0.819	0.03	0.601	3.109	0.11	0.772
Tea	4.030	0.061	0.133	10.172	0.423	**<0.001**
Alcohol	6.086	0.076	**0.041**	4.313	0.131	0.639
Smoking	2.408	0.046	0.312	9.531	0.263	**0.036**
Sweets	0.915	0.029	0.632	1.557	0.102	0.818

Bold: *p* < 0.05.

## Data Availability

The datasets generated and analyzed during the current study are available from the corresponding author on reasonable request.

## Supplementary Materials

The following supporting information can be downloaded at: https://www.mdpi.com/article/10.3390/healthcare10050833/s1, Supplementary File S1: Survey questionnaire.
