# Supplementary material for: Helicobacter pylori Infection in Croatian Population: Knowledge, Attitudes and Factors Influencing Incidence and Recovery
Source: Healthcare (Basel). 2022 Apr 30;10(5):833. doi: 10.3390/healthcare10050833 (PMC9141647; doi:10.3390/healthcare10050833)
Supplement: Supplementary file 1 [file healthcare-10-00833-s001.zip › healthcare-1680114-supplementary.pdf]

Supplementary file S1: Survey questionnaire

1. Basic information

1. What is your age?

19-34 years

35-60 years

>60 years

2. What is your sex?

Female

Male

3. What is your educational level?

Primary education (and below)

Secondary education

Specialty

Bachelor's degree

Master's degree (and above)

4. Where do you live at present?

Urban

Suburban

Rural

5. What is your occupation category?

Chief executives, senior officials and legislators

Science and engineering professionals

Technicians and associate professionals

Clerical support workers, service and sales workers

Skilled agricultural, forestry and fishery workers

Plant and machine operators, and assemblers

Student

Other

6. Do you have the following habits?

Long-term alcohol	Yes	No
Long-term smoking	Yes	No
Long-term high-fat diet	Yes	No
Long-term sweets	Yes	No
Long-term seafood	Yes	No
Long-term preserved food	Yes	No
Long-term coffee	Yes	No
Long-term tea	Yes	No

7. Have you ever heard of *Helicobacter pylori (H. pylori)?*

Yes

No

2. Basic knowledge of *H. pylori*

1. Is *H. pylori* infectious?

Yes
No
Do not know

2. Could *H. pylori* cause gastritis?

Yes
No
Do not know

3. Could *H. pylori* cause stomach cancer?

Yes
No
Do not know

4. Would it be meaningful to reduce the number of *H. pylori* infections?

Yes
No
Do not know

5. Would the reduction of *H. pylori* infections lower stomach cancer incidence?

Yes
No
Do not know

3. Attitudes towards *H. pylori* testing and therapy and sources of information

1. Have you ever been screened for H*. pylori* infection?

Yes

No

If you chose **No** as an answer of the first question, answers the questions 2 and 3

2. Reason for not being screened

It is not included in hospital physical examination

There are no obvious symptoms and I don't want to check them

I am young and not necessary to test it

I am old and worry about the risk of screening

Other

3. Support for *H. pylori* screening

Yes

No

Neutrality

If you chose **Yes** as an answer of the first question, answers the questions 3 to 8

4. Which of the following methods would you choose to screen for *Helicobacter pylori*?

Go and test *H. pylori* on my own

Routine medical examinations when available

Not clear

5. If you are infected with *Helicobacter pylori*, would you be advised to screen your family for *Helicobacter pylori*?

Yes

No

Neutrality

6. What is your attitude towards *H. pylori* screening?

No need to check

Neutrality

Supporting screening

7. If you are infected with *Helicobacter pylori*, will you eradicate it?

Yes

No

Neutrality

8. If you are negative for *Helicobacter pylori* but your family is positive for *Helicobacter pylori*, do you recommend your family to eradicate it?

Yes

No

No opinion

9. How did you get the relevant information about *Helicobacter pylori*?

A Television or broadcasting

B. Newspapers and magazines

C. Friends/family

D. Medical examinations

E. Networks

F. Books

G. Other modalities

<u>5. Personal experience with *H. pylori* infection</u>

1. Have you ever been infected with *H. pylori*?

Yes

No

Not clear

2. If you had an infection, did you receive a treatment?

Yes

No

Not clear

If you chose **No** as an answer of the second question, answers the question 3

3. What is the reason why you have been infected with *Helicobacter pylori* but have not eradicated it?

Carrying H. pylori has no impact on health and life

Economic factors

Worry about side effects of drug

Fear of relapse after eradication

Other

If you chose **Yes** as an answer of the second question, answers the questions 4 to 13

4. Have you ever received a regimen including antibiotics?

Yes

No

Not clear

5. Have you received triple/quadruple eradication treatment?

Yes

No

Not clear

6. What was the duration of your treatment?

<7 days

7-10 days

11-14 days

>14 days

Not sure

7. Did you have a re-examination after eradication treatment?

Yes

No

Not sure

Did not answer

8. Have you been re-infected after the treatment?

Yes

No

Not sure

9. Have you had any of the following adverse reactions during taking the medicine?

Abdominal pain

Diarrhea

Dry mouth

Constipation

Other

No adverse drug reactions

What is your evaluation of the previous treatments' results?

10. Estimate symptoms' improvement after the treatment:

Deterioration

No change

Slight improvement

Improvement

Complete improvement

11. Worry caused by treatment

All the time

Most of the time

Sometimes

Occasionally

Never

12. I considered other treatment options due to unsatisfactory results of the original therapy:

All the time

Most of the time

Sometimes

Occasionally

Never

13. Evaluation of patients' quality of life

Do you still have the following symptoms after treatment?

**Abdominal pain regardless of intensity**	Very often	Often	Sometimes	Almost never	Never
**Stomach (upper abdomen) fullness**	Very often	Often	Sometimes	Almost never	Never
**Belching or flatulence**	Very often	Often	Sometimes	Almost never	Never
**Vomiting**	Very often	Often	Sometimes	Almost never	Never
**Nausea**	Very often	Often	Sometimes	Almost never	Never
**Heartburn**	Very often	Often	Sometimes	Almost never	Never
**Bitter taste**	Very often	Often	Sometimes	Almost never	Never
**Lack of appetite**	Very often	Often	Sometimes	Almost never	Never
**You must give up eating some favorite food due to illness**	Very often	Often	Sometimes	Almost never	Never
**Be dissatisfied with your life**	Very often	Often	Sometimes	Almost never	Never
**Situation affecting the continuation of daily amateur activities**	Very often	Often	Sometimes	Almost never	Never
**The relationship with your relatives and friends is affected due to illness**	Very often	Often	Sometimes	Almost never	Never
**Restrictions on sexual life**	Very often	Often	Sometimes	Almost never	Never
**Insomnia**	Very often	Often	Sometimes	Almost never	Never
**Illness has forced you to adopt a separate diet**	Very often	Often	Sometimes	Almost never	Never
**Feel sad because of your illness**	Very often	Often	Sometimes	Almost never	Never
**Frustrated by your illness**	Very often	Often	Sometimes	Almost never	Never
**Feel nervous or afraid due to illness (such as fear of canceration, etc.)**	Very often	Often	Sometimes	Almost never	Never
